# Humans flexibly use visual priors to optimize their haptic exploratory behavior

**DOI:** 10.1038/s41598-024-65958-6

**Published:** 2024-06-28

**Authors:** Michaela Jeschke, Aaron C. Zoeller, Knut Drewing

**Affiliations:** Experimental Psychology, Justus-Liebig University, 35390 Gießen, Germany

**Keywords:** Neuroscience, Physiology, Psychology

## Abstract

Humans can use prior information to optimize their haptic exploratory behavior. Here, we investigated the usage of visual priors, which mechanisms enable their usage, and how the usage is affected by information quality. Participants explored different grating textures and discriminated their spatial frequency. Visual priors on texture orientation were given each trial, with qualities randomly varying from high to no informational value. Adjustments of initial exploratory movement direction orthogonal to the textures’ orientation served as an indicator of prior usage. Participants indeed used visual priors; the more so the higher the priors’ quality (Experiment 1). Higher task demands did not increase the direct usage of visual priors (Experiment 2), but possibly fostered the establishment of adjustment behavior. In Experiment 3, we decreased the proportion of high-quality priors presented during the session, hereby reducing the contingency between high-quality priors and haptic information. In consequence, even priors of high quality ceased to evoke movement adjustments. We conclude that the establishment of adjustment behavior results from a rather implicit contingency learning. Overall, it became evident that humans can autonomously learn to use rather abstract visual priors to optimize haptic exploration, with the learning process and direct usage substantially depending on the priors’ quality.

## Introduction

Haptic perception is an inherently active process. Humans typically adjust their exploratory behavior to the current task and present objects to achieve optimal perceptual performance and to behave most efficiently^1–3^. Closed-loop processes based on sensory signals partly enable this motor adaptation: Sensory information is gathered and integrated over the sequential exploration process and used for movement adaptation^4,5^. For instance, when trying to judge the ripeness of an avocado, you are likely to adjust your executed force after each indentation to maximize the intake of sensory information about the fruits’ compliance while trying not to squish it too harshly. In many situations, humans do not only rely on their gathered haptic information but additionally have sources for prior information about the objects they are going to interact with: Prior information can e.g. arise from recent interactions with similar objects, be semantic (for instance, verbal), or stem from visual input^1,6,7^. Regarding motor control in general, such prior information can help to adjust motor behavior already before and during initial object contact^8^. Previous studies could show for example that participants incorporated prior information about weight in their lifting and grasping behavior^6^. For exploratory behavior specifically, studies demonstrated that participants explored stimuli with higher initial peak forces when they expected harder as compared to softer stimuli^7^ or when they expected lower differences between the stimuli’s compliances^1^. Prior information hence seems to play a role for (open-loop) haptic exploratory movement control as well. For visual information in particular, findings are inconclusive so far: it has been observed that humans adapt their initial indentation force in reaction to explicit visual information on a stimulus’ compliance; but this adaptation did not resemble the same tuning behavior as evoked by other predictive or sensory signals^7^. The authors suspected that explicit and implicit prior information lead to different influences on exploratory behavior, which could explain the conflicting research evidence. The current study thus extends previous findings by systematically investigating whether humans use rather abstract, i.e. implicit prior visual information for optimizing their subsequent haptic exploration, elucidating factors that modify the usage, and examining the flexibility of the process. For that, we assess the usage of prior visual information on texture orientation for adjustment of initial movement direction.

Haptic texture exploration is typically performed by moving the fingertips laterally across an object’s surface for multiple times^3^. This produces small patterns of vibrations, i.e. temporal cues, that enable humans to discriminate fine textures by their microgeometry^9^, and spatial cues that are based upon spatial variability in skin deformation by the texture. Spatial cues have been shown to dominate the perception of coarse textures^10^. To investigate texture exploration in psychophysical experiments, groove/ridge gratings are being widely used. Those consist of periodically repeating grooves and ridges, often arranged with a concise orientation. Their roughness is mostly affected by changing inter-element spacing (groove width), element width (ridge width) or their spatial frequency^11^; with increased roughness perception, the larger the separation is. The intake of temporal cues during exploration of these gratings can be maximized by moving orthogonal to the gratings’ orientation. Lezkan and Drewing^12^ showed that indeed, movement direction in spatial frequency discrimination is optimized accordingly: in their experiment, participants had to haptically discriminate grating pairs and the movement data revealed that they adapted the directions of their final finger movements orthogonal to the texture orientations. This implies that they exhibited adaptation behavior based on sensory information gathered over the course of the exploration. In a follow-up experiment, this adaptation behavior was found to be beneficial in terms of perceptual precision. In the current study, we used a similar method and examined whether visual priors on grating orientation, presented before each trial, evoke similar adjustments of movement direction as in^12^, but, importantly, already at initial contact. We focused on the initial movements’ direction as it can be assumed to be hardly affected by haptic sensory feedback. Thus, it is a suitable indicator of the usage of the visual priors.

**Figure 1:**
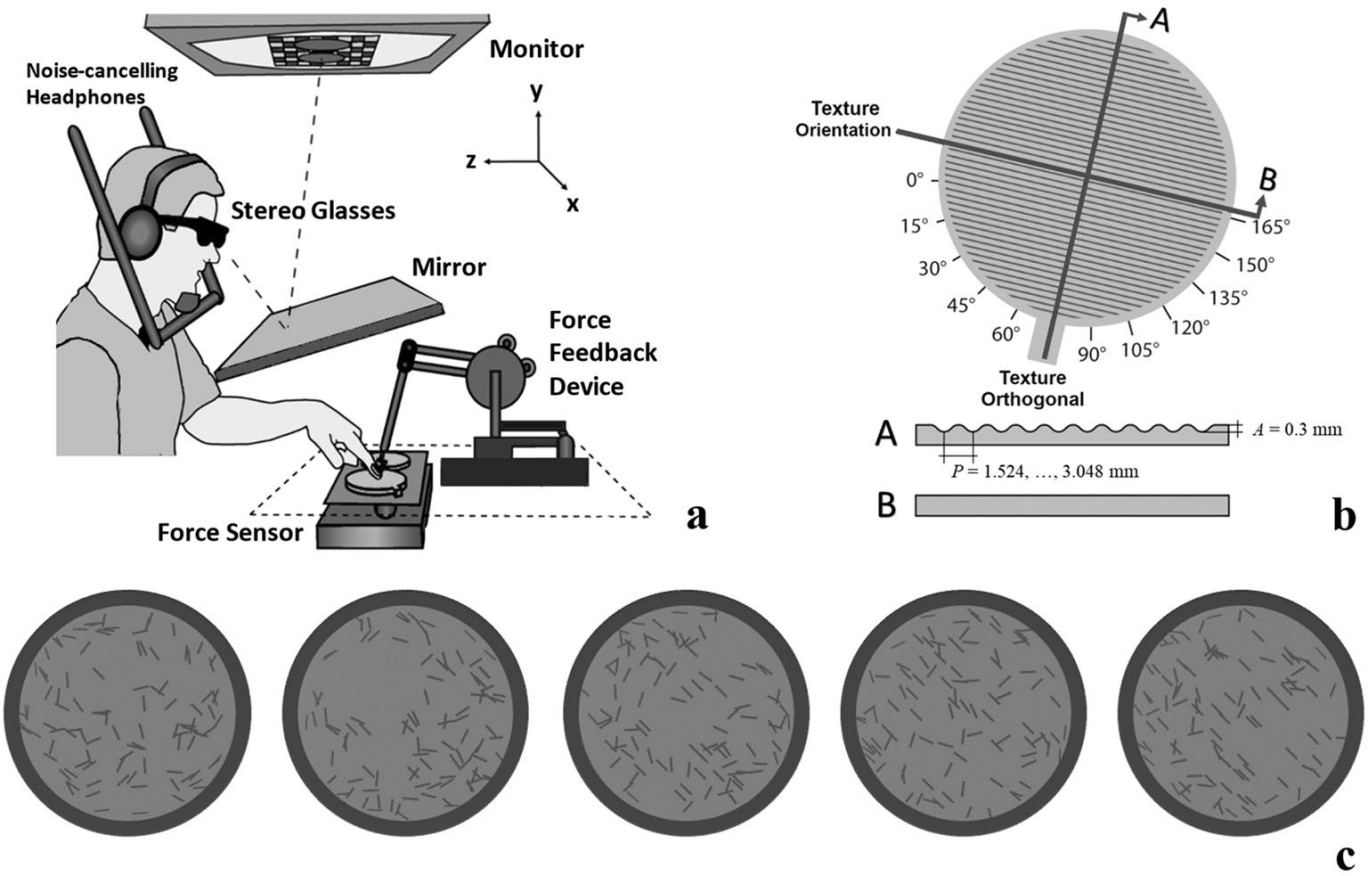
Setup and stimuli. (**a**) visuo-haptic workbench (**b**) haptic stimulus in orientation 165° **c.** examples of visual priors with qualities 0%, 15%, 25%, 35%, and 50% (from left to right) in orientation 135°. Based on pilot studies in which participants judged the orientation of those priors, we manipulated their qualities by varying the percentage of line segments following the same orientation as the gratings’ ridges while the remaining ones were randomly oriented.

The role of information quality for the priors’ usage was in the focus of Experiment 1. Note that most results of this experiment have been pre-published in a conference paper^13^ and are being reinvestigated with further experiments in the current study. Oftentimes, sensory input is noisy, lossy, or unreliable due to e.g., memory decay, attentional processes, or physical conditions of the environment. Many researchers have addressed the question on how the human nervous system combines and integrates different sources of (multi)sensory information and what role their reliability (inverse variance) plays in this context^14–19^. They demonstrated that the brain often functions similar to a maximum-likelihood integrator, combining different sources of sensory information by weighting them according to their reliability^14–16^. Thus, humans maximize the information they gather from their environment, reducing perceptual errors and negative behavioral consequences resulting from wrong decisions. Bayesian models for multisensory integration additionally take prior information into account, which is again weighted by its’ reliability. They have been successfully applied to many sensorimotor problems^20,21^. Based on these frameworks and findings on motor control we presumed that the usage of visual priors for exploratory movement adjustments might also depend on their quality; quality here refers to the level of precision at which information can be derived from the visual input. As haptic exploration has been demonstrated before to be an inherently adaptive process^1–3^, it seems plausible that humans also take the quality of prior information into account when using it for movement preparation. To test this, we presented visual priors that randomly differed in their quality each trial, ranging from high to no informational value, and indicated the orientation of upcoming haptic gratings (Fig. 1c). We aimed to artificially induce quality variations in the priors to imitate abovementioned real-world influences such as e.g. movement artifacts, insufficient illumination, low resolution of an image, or deficient visual acuity of an observer. We hypothesized that the presentation of informational visual priors leads to movement adjustments at initial haptic contact and expected stronger movement adjustments towards orthogonal when individuals receive priors of higher as compared to lower quality. This behavior was well reflected in the data, but not yet fully established in the first quarter of the session, suggesting that some learning is required first.

In Experiment 2, we further investigated the flexibility with which the priors are used by examining another potential moderator of the adaptation process: the task demands. Task demands have been demonstrated to affect participants’ exploratory process in other contexts: Participants executed higher forces when indenting stimulus pairs which had smaller differences in their compliance^1^. Moreover, findings in the field of vision research suggest that task demands shape the execution of eye movements: The size of the functional visual field was shown to shrink with increasing task demands during visual search^22^ and observers seemed to prefer strategies facilitating efficient scanning when being confronted with more difficult tasks^23^, cf.^24^. Here, we asked whether task demands influence the usage of visual priors for optimization of haptic exploratory behavior by implementing two levels of task demand, which were established implicitly by presenting trials of the same level in one block. Hence, an effect of task demand on prior usage would be caused by a predictive process: Individuals would expect a certain task demand for the upcoming trial and possibly adjust their subsequent behavior accordingly. The presentation order of the blocks was counterbalanced between participants. For “high demand” trials, stimulus pairs had smaller differences between their spatial frequencies and thus were more difficult to discriminate than “low demand”- pairs. For economic reasons, we only incorporated three (equidistant) levels of visual quality. We hypothesized that higher demands lead to higher extents of adjustment behavior in reaction to the priors; resembling a compensation mechanism for the increased effort that is needed to interpret the haptic sensory signals while trying to achieve a satisfying performance^25^. However, results revealed no increase of initial movement adjustments when task demands were higher, suggesting that task demands do not have a rapidly modulating predictive effect on the direct usage of the priors*.* Higher demands were however associated with other typical compensation mechanisms such as prolonged exploration durations. Notably, we observed a trend suggesting that certain initial learning conditions, i.e. higher task demands in the initial experimental block, might foster the establishment of movement adjustments and their prevalence throughout the session.

In Experiment 3 we aimed to explore the flexible nature of the observed adaptation behavior from a different perspective: Instead of assessing the direct usage of the priors, we here focused on the establishment of the adjustments. For that, we investigated the role of the priors’ visual quality during establishment of adjustment behavior and the mechanism that underlies the establishment. Based on the experience-dependent effects on movement adjustments observed in the previous experiments, we speculated that a form of contingency learning might enable the adjustment behavior. “Contingency” describes a conditional probabilistic relation between two events/stimuli; many studies have demonstrated that humans can detect and use contingency to make predictions and infer causal relationships^26^: high probabilities of an event B given Event A enhance learning^26^, while lower probabilities, i.e. lower contingencies, can hinder the learning process^27^. Lower-quality visual priors in our study indicate less precisely the orientation of an upcoming haptic texture and hence yield lower predictability for the following orientation. Results from the first experiment confirm this, as the low-quality prior (15%) did not produce substantial adaptation behavior. Hence, we consider less frequent presentations of medium-to high quality priors (and more frequent lower-quality priors) as a reduced contingency of the visual priors’ orientation and the haptic stimulus orientation and tested whether this affects the learning of the pairing and consequently the establishment of movement adjustments. As influences on the establishment of prior usage can only be studied in between-participant designs, the sample of Exp. 2 served as the first group (high contingency, 0%, 25%, 50% priors) and the sample of the following Exp. 3 as the second group (low contingency; 0%, 15%, 50% priors). Hence, we used the same experimental procedures in both experiments. We hypothesized that the extent of adjustment behavior will be substantially reduced with low as compared to high contingency. Indeed, participants in the low-contingency group exhibited almost no adjustment behavior anymore; even when prior quality was high. Not only the direct prior usage, but also the learning process hence seems to crucially depend on the priors’ quality. Taken together, the results from the three experiments suggest that optimization behavior is implicitly established when deemed sufficiently useful, and the direct usage of priors is flexibly adjusted.

## Results

**Figure 2:**
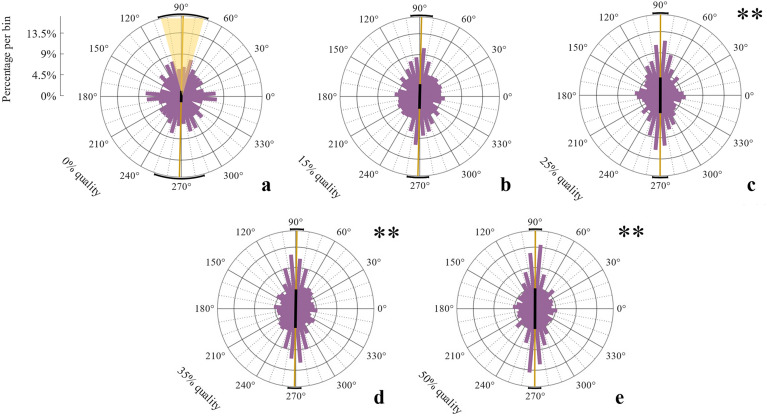
Experiment 1: Initial movement direction histograms for each quality condition including all participant data. Bin size: 10°. Textures were aligned to a 0° orientation. Possible movement directions varied only between 0–180° and were mirrored on the lower part of each figure. Orange lines indicate mean directions, black bars in the center of each circle represent resultant vector lengths. Black circle segments mark the 95% confidence interval of the mean direction. Yellow cone in **a** marks the 90° ± 15° area of movement directions.

In Experiment 1, we studied the role of information quality for the priors’ usage in haptic frequency discrimination^13^. Participants spent on average 5.5 s (*SD* = 1.36) per trial on the stimuli, performed 6.6 strokes (*SD* = 2.67), switched 1.7 times (*SD* = 0.37) between them and gave 91.1% correct responses (*SD* = 8.88%). Correct responses did not depend on the priors’ Visual Quality; *F*(4,60) = 0.18, *p* = 0.95, η^2^_*p*_ = 0.03 in repeated-measures ANOVA of arcsine-transformed percentages (i.e., the arcsines of their square-roots). Likewise, movement variables such as number of strokes, switches between stimuli, or time spent on stimuli did not differ between quality conditions (all *p*s > 0.12). We plotted angular distributions of movement directions of all initial movements (in the following: “strokes”) separately for each visual quality condition (Fig. 2a–e). Bonferroni-corrected V-tests on deviations from uniformity were performed on distributions of individual average movement directions per quality condition, and separately for the first, middle, and last strokes. For initial strokes, results indicated movement adjustments towards orthogonal for the 25%, 35% and 50% quality conditions, but not for the 0% and 15% quality condition (0%: *V* = 7.26, *p* = 0.08, 15%: *V* = 6.56, *p* = 0.18, 25%: *V* = 11.71, *p* < 0.001, 35%: *V* = 9.54, *p* < 0.001, 50%: *V* = 9.15, *p* < 0.001). For individual average movement directions of middle and last strokes in a trial (not depicted), distributions of all quality conditions deviated significantly towards orthogonal (all *p* < 0.001, all *V* > 11.37).

**Figure 3:**
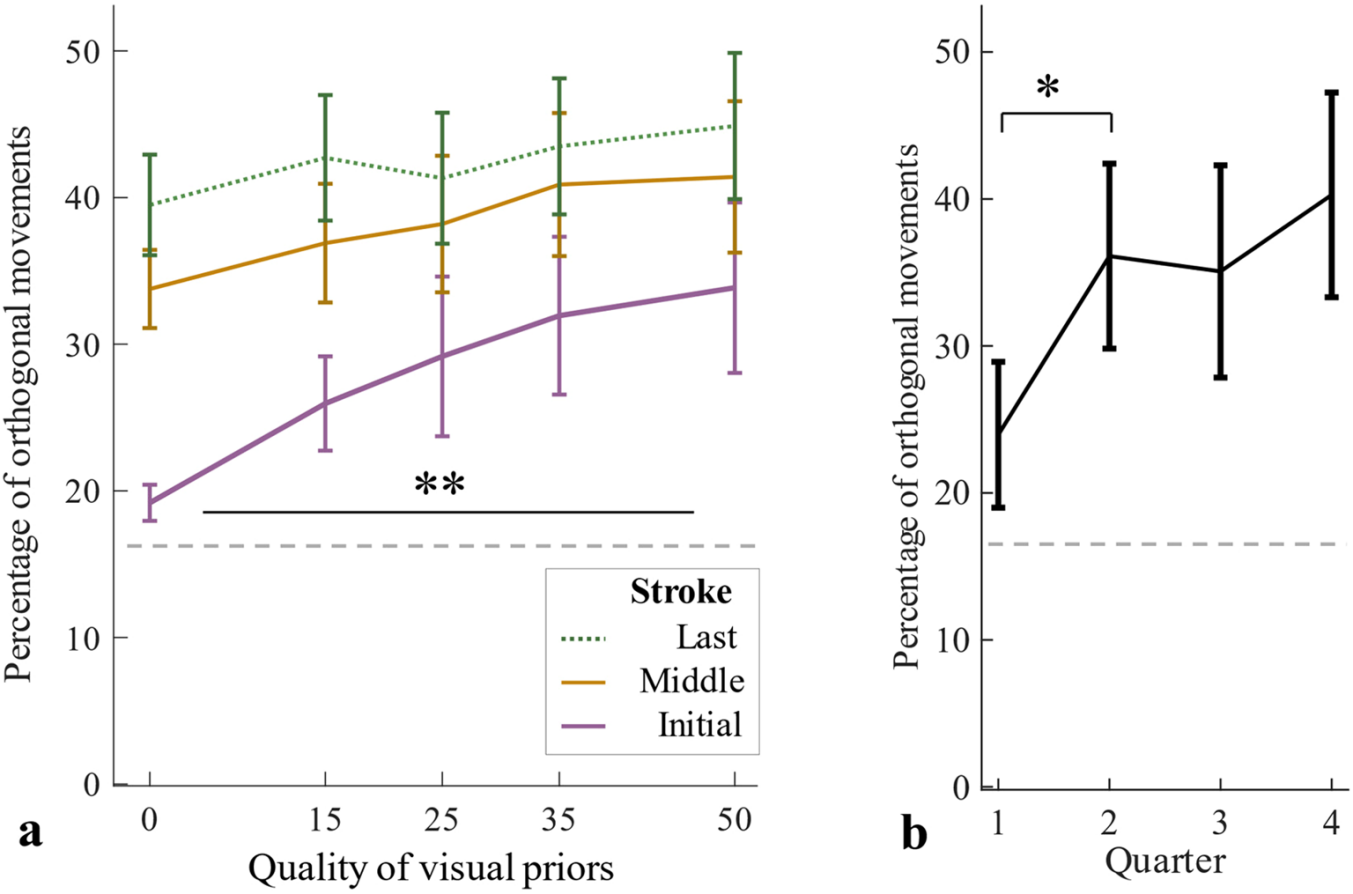
Experiment 1: Percentages of orthogonal movements. (**a**) Average percentages of orthogonal movements and standard errors for each quality condition and movement type (**b**) Percentages of initial orthogonal movements for trials of highest quality (50%) for each chronological quarter of the experiment. The dashed grey line represents chance level (entirely uniform movement distribution, 16.67%).

Proportions of movements close-to- orthogonal to the textures’ surface 90° (± 15°) (see Fig. 2a) among all initial strokes across participants (Fig. 3a) entered an ANOVA with the within-participant factor Visual Quality. As expected, proportions of orthogonal initial strokes were higher with higher visual qualities, *F*(4, 60) = 4.39, *p* = 0.036, η^2^ = 0.23. The proportional increase of orthogonal strokes in reaction to priors of increased visual quality was confirmed by a linear trend, *F*(1,15) = 6.11, *p* = 0.026, η^2^ = 0.29, analyzed with a linear contrast on the proportions of orthogonal strokes across the quality conditions. For middle and last strokes, that is after gathering sensory information on grating orientation, proportions of orthogonal strokes did not differ between quality conditions, *F*(4,60) = 2.33, *p* = 0.11, η^2^ = 0.14, and *F*(4,60) = 1.52, *p* = 0.28, η^2^ = 0.09, respectively. Same patterns were found for the individual mean resulting vector lengths of initial, middle and last strokes, being indices of variance in movement direction, which also entered ANOVAs with the within-participant factor Visual Quality (Supplementary Section I, Fig. S1), corroborating the evidence. Finally, we observed that prior usage was established during the beginning of the experiment; proportions of orthogonal initial strokes for trials with high visual quality (50%) entered an ANOVA with the within-participant factor Experimental Quarter (Fig. 3b). There was a significant main effect, *F*(3,45) = 4.92, *p* = 0.005, η^2^_*p*_ = 0.25, with proportions of the first quarter of the session being smaller than those of the second quarter, as Bonferonni-corrected post-hoc tests revealed, *t*_*15*_ = 2.73, *p* = 0.02 (three tests, adjacent levels compared, other *p*s > 0.50). In the questionnaire, only two participants reported that they were aware about the visual priors indicating the orientation of the upcoming haptic stimulus and that they intentionally adjusted their movements accordingly, i.e. by moving orthogonal to the indicated orientation. All others responded “no” to the first question (i.e., whether they actively paid attention to the visuals).

**Figure 4:**
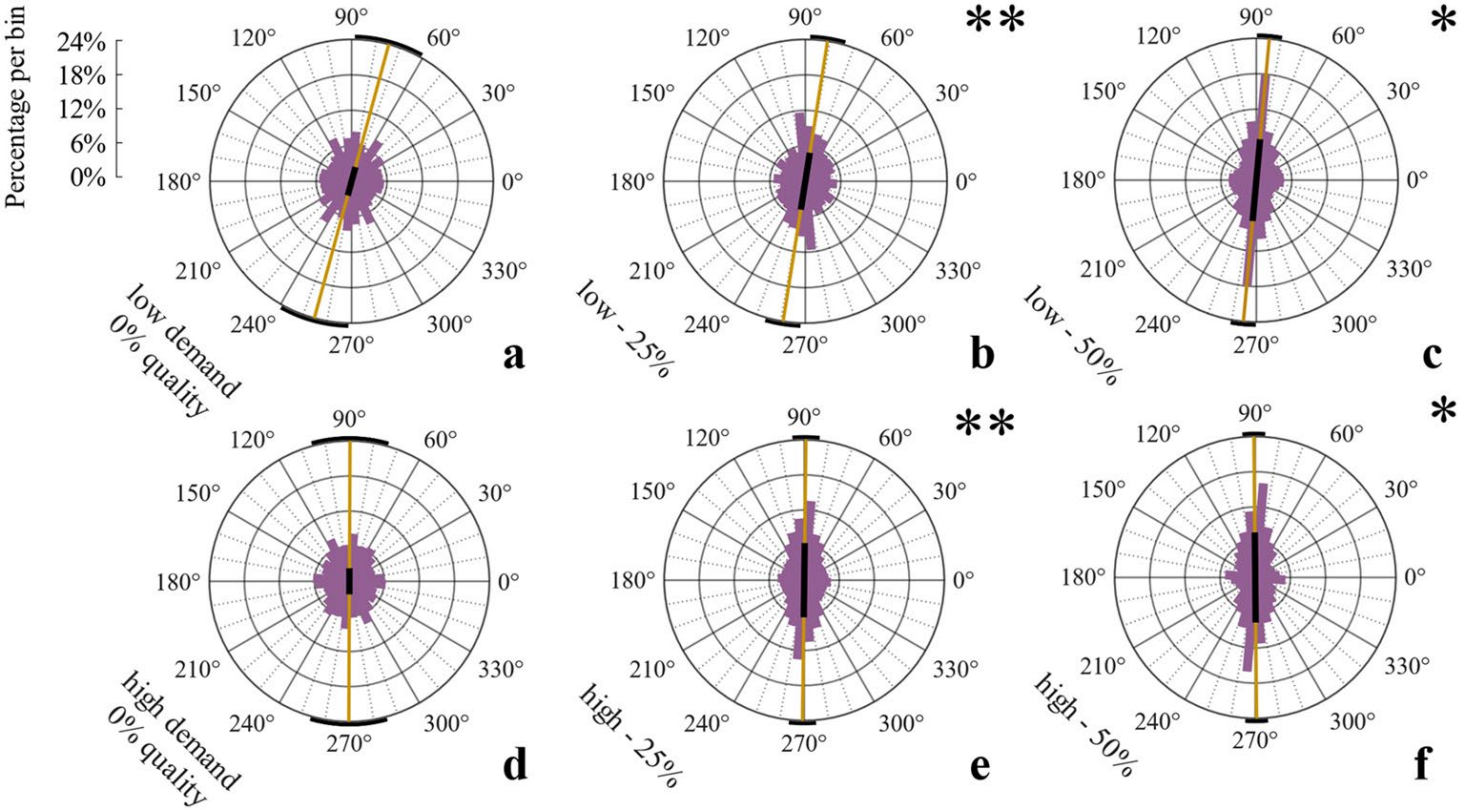
Experiment 2: Initial movement direction histograms for each quality*demand condition including all participant data.

In Experiment 2, we studied the role of task demands. Participants spent on average 10.85 s (*SD* = 3.16) per trial on the stimuli, performed 14.78 strokes (*SD* = 5.74), switched 2.48 times (*SD* = 0.68), and gave 90.2% correct responses (*SD* = 7.09%). Participants executed more strokes during more demanding trials as compared to less demanding ones, *t*(1,17) = 2.89, *p* = 0.011, *d* = 0.36, switched more often, *t*(1,17) = 3.55, *p* = 0.002, *d* = 0.54, and spent more time on the stimuli, *t*(1,17) = 2.64, *p* = 0.017, *d* = 0.50; but kept their response accuracy constant, *t*(1,17) = 0.74, *p* = 0.47, *d* = 0.02. Again, none of those variables differed between visual quality conditions (all *p* > 0.08). We plotted angular distributions of movement directions of initial strokes separately for each visual quality condition and the two demand conditions (Fig. 4a–f). Bonferroni-corrected V-tests on deviations from uniformity were performed on the distributions of individual average movement directions per quality and demand condition, and separately for initial, middle, and last strokes. For initial strokes of both the low and high demand condition, significant tests indicated adjustments towards orthogonal movement for the 25% and 50% quality conditions, but not for the 0% condition (low demand: 0%: *V* = 7.55, *p* = 0.09, 25%: *V* = 10.18, *p* < 0.001, 50%: *V* = 8.96, *p* = 0.015, high demand: 0%: *V* = 6.70, *p* = 0.27, 25%: *V* = 11.65, *p* < 0.001, 50%: *V* = 11.64, *p* = 0.015. For middle and last strokes, distributions of all quality and demand conditions significantly indicated adjustment towards orthogonal (all *p* < 0.001, all *V* > 14.28).

**Figure 5:**
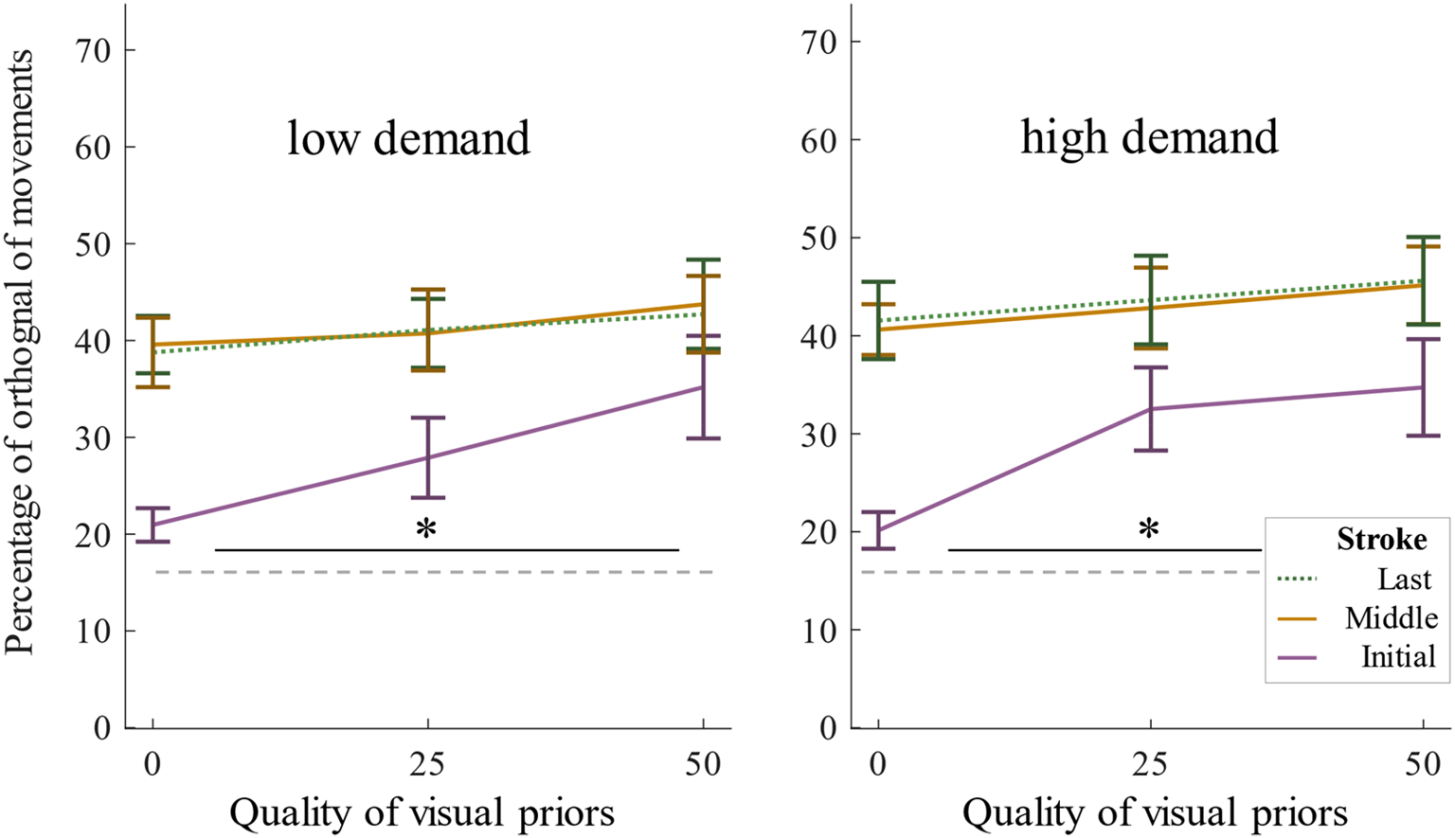
Experiment 2: Average percentages of orthogonal movements and standard errors for each quality condition and movement type, for each demand condition separately.

**Figure 6:**
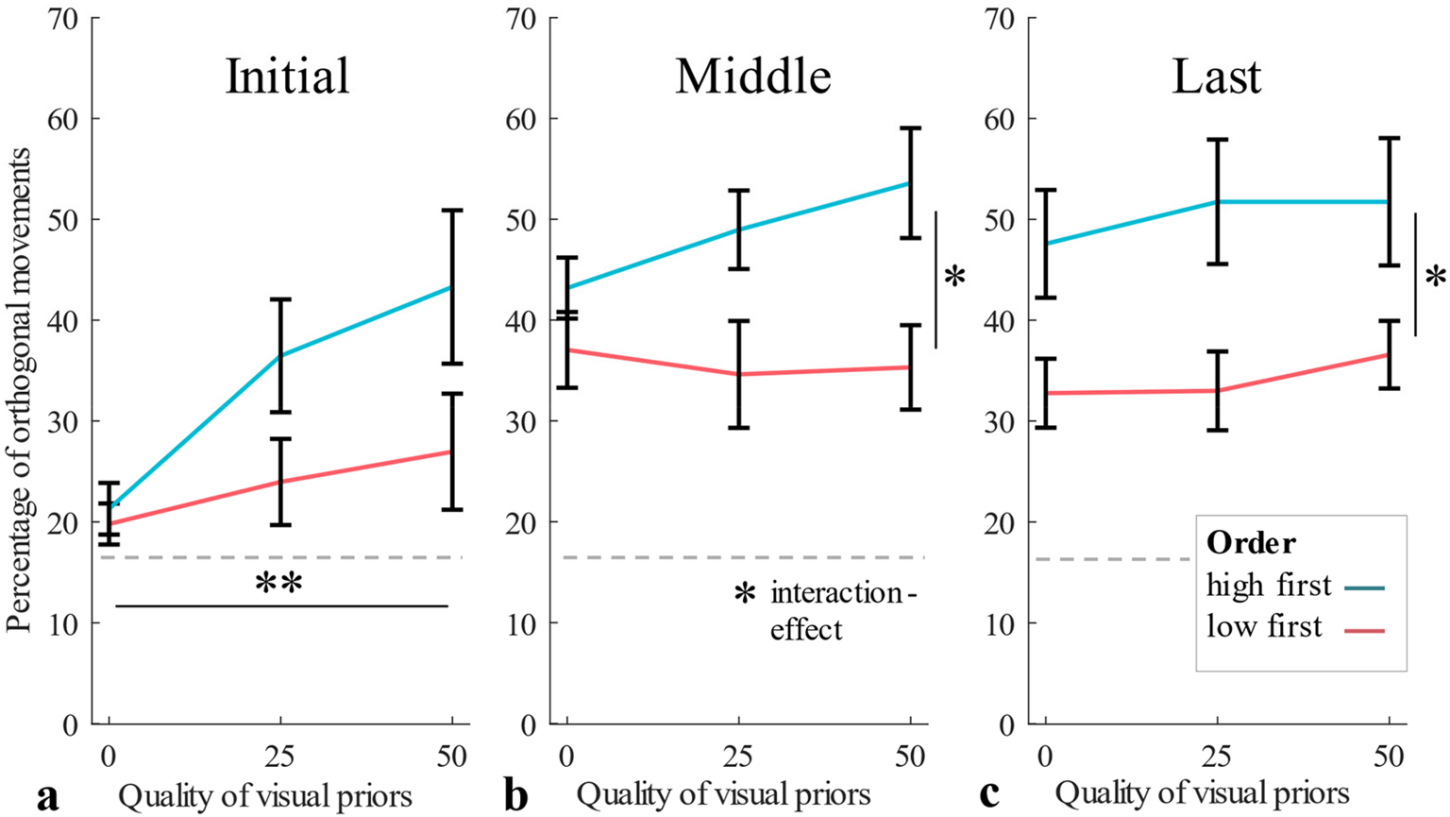
Experiment 2: (**a**) Average percentages of orthogonal initial movements and standard errors for each quality condition of participants starting with low demand block vs. high demand block (**b**) percentages for middle movements **c.** percentages for last movements.

Proportions of orthogonal initial strokes entered a three-way mixed-design ANOVA with the within- participants factors Visual Quality (0%, 25%, 50%) and Task Demand (high vs. low) (Fig. 5) and the between-participants factor Order (participants that started the experiment with the low demand-block vs. those that started with the high demand-block). The proportion of orthogonal initial strokes was again higher with higher visual qualities, *F*(2,32) = 6.83, *p* = 0.014, η^2^_*p*_ = 0.30), confirmed by a linear trend, *F*(1,17) = 7.13, *p* = 0.016, η^2^_*p*_ = 0.30. There was no main effect of Task Demand, *F*(1,16) = 0.33 , *p* = 0.57, η^2^_*p*_ = 0.02. The main effect of Order, *F*(1,16) = 3.67, *p* = 0.073, η^2^_*p*_ = 0.19, marginally failed to reach significance. This trend might though suggest that participants show stronger adjustments when starting with high demand trials as compared to starting with low demand trials (Fig. 6a). None of the three possible interaction effects were significant (all *p* > 0.16). Proportions of close-to-orthogonal movements among middle and last strokes entered three-way mixed-design ANOVAs with the within-participants factors Visual Quality and Task Demand (see Fig. 5) and the between-participant factor Order (Fig. 6b,c). For middle strokes, there was no main effect of Visual Quality, *F*(2,32) = 2.46, *p* = 0.10, η^2^_*p*_ = 0.13 or Task Demand, *F*(1,16) = 1.04, *p* = 0.32, η^2^_*p*_ = 0.06, but a main effect of Order, *F*(1,16) = 5.09, *p* = 0.038, η^2^_*p*_ = 0.24, suggesting stronger adjustments of later movements when participants were initially confronted with the more demanding block. The interaction effect of Quality and Order was significant, *F*(2,32) = 4.93, *p* = 0.014, η^2^_*p*_ = 0.23, moderating the magnitude but not the direction of the Order-effect (other interaction effects all* p* > 0.27). For last strokes, only the main effect of Order reached significance, *F*(1,16) = 6.01, *p* = 0.026, η^2^_*p*_ = 0.27 (all other *p* > 0.06). Proportions of orthogonal initial strokes of trials with high visual quality entered a one-way repeated measures ANOVA with the within-participants factor Quarter, which gave no significant result, *F*(3,51) = 0.954, *p* = 0.392, η^2^_*p*_ = 0.05 (Quarter 1: *M* = 30.93, *SD* = 17.71, 2: *M* = 36.00 , *SD* = 22.87, 3: *M* = 35.78, *SD* = 26.69, 4: *M* = 37.77, *SD* = 29.55).

In Experiment 3, we replicated Exp. 2 but with a reduced contingency between high-quality visual priors and haptic information to study establishment of motor adjustment. Here, participants spent on average 6.23 s (*SD* = 2.02) per trial on the stimuli, performed 8.36 strokes (*SD* = 3.74), switched 2.03 times (*SD* = 0.49) and gave 87.1% correct responses (*SD* = 6.48%). Again, participants executed more strokes during more demanding trials, *t*(1,13) = 2.45, *p* = 0.03, *d* = 0.36, spent more time on the stimuli, *t*(1,13) = 2.66, *p* = 0.02, *d* = 0.50, but kept their response accuracy constant, *t*(1,13) = 0.70, *p* = 0.50, *d* = 0.02.

**Figure 7:**
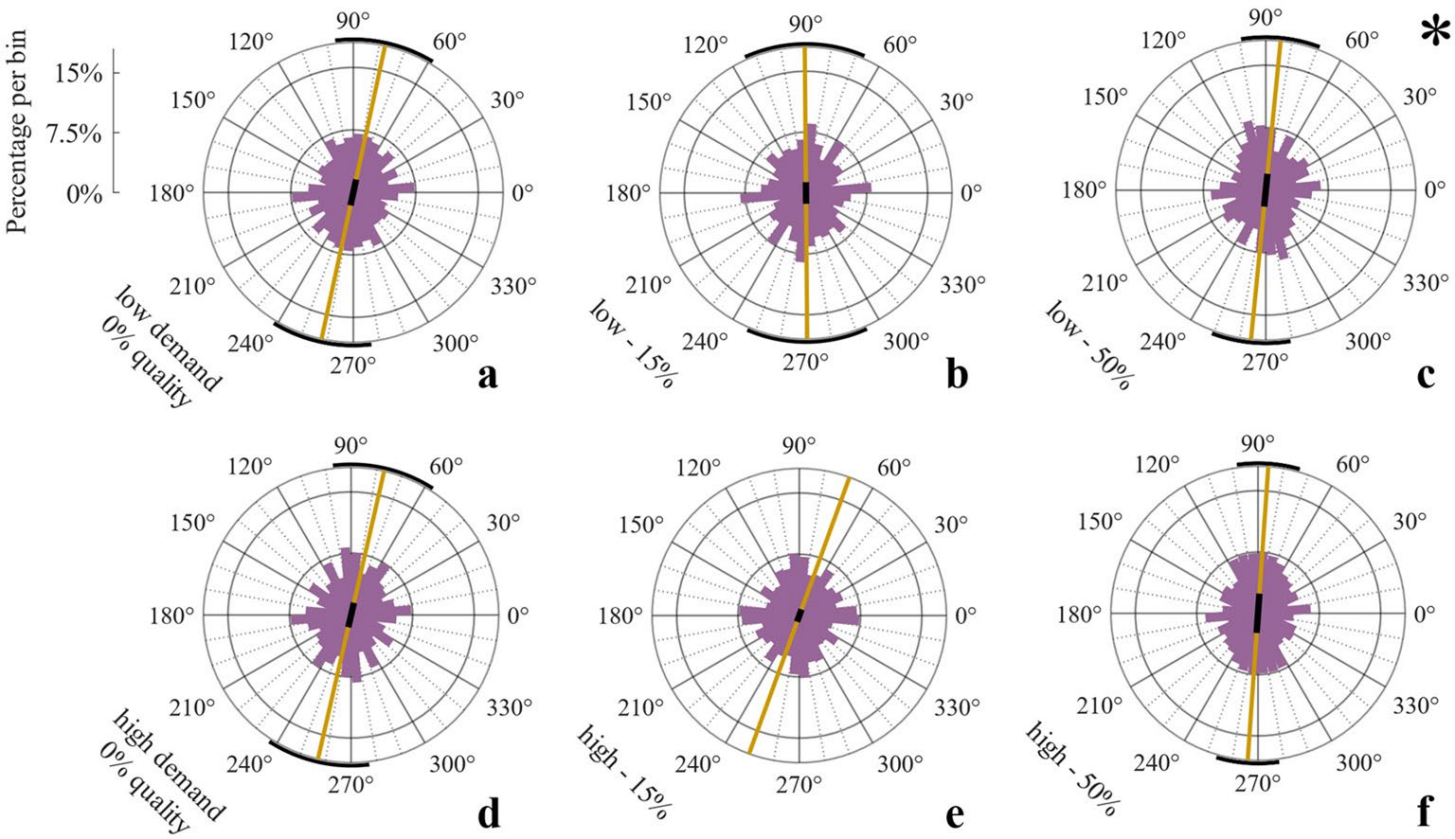
Experiment 3: Initial movement direction histograms for each quality*demand condition including all participant data.

In V-tests, distributions of initial strokes did not significantly deviate from uniformity in most conditions, suggesting hardly any optimization of movement behavior (high demand, 0%: *V* = 4.82, *p* = 0.51, 15%: *V* = 3.02, *p* = 1, 50%: *V* = 6.88, *p* = 0.075, low demand: 0%: *V* = 6.27, *p* = 0.135, 15%: *V* = 2.37, *p* = 1, exception 50%: *V* = 7.53, *p* = 0.033, Fig. 7). This is strongly distinct from the movement adjustments observed in Exp. 2 with higher contingency. For individual average movement directions of middle and last strokes, again all distributions deviated significantly from uniformity as had been the case in Exp. 2 (all *p* < 0.001, all *V* > 11.65).

**Figure 8:**
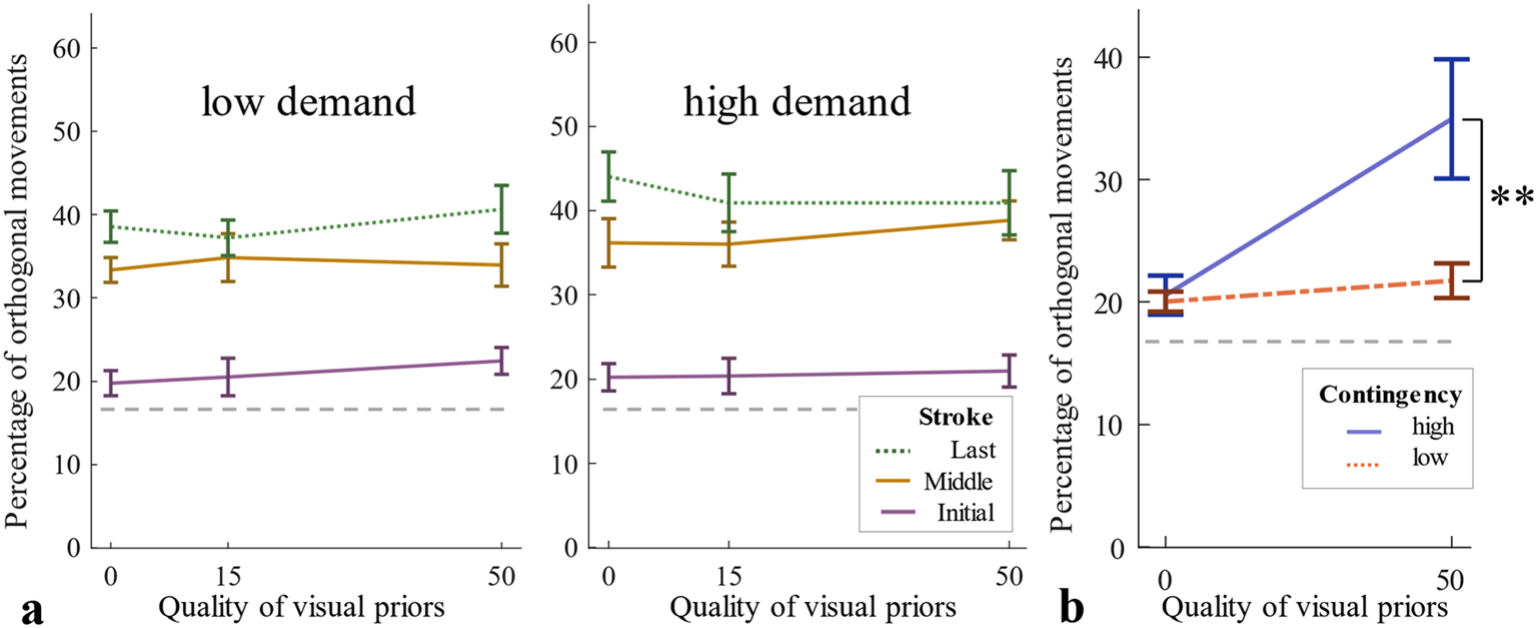
Experiment 3: (**a**) Average percentages of orthogonal movements and standard errors for each quality condition and movement type, for each demand condition separately (**b**) Average percentages of initial orthogonal movements for 0% and 50% visual quality trials of Experiment 2 (high contingency) and 3 (low contingency).

In line with this lack of initial adjustment, we did not find significant effects in proportions of orthogonal initial strokes neither in a three-way mixed-design ANOVA with the within-participant factors Visual Quality and Task Demand and the between-participants factor Order, nor when analyzing adjustment in different quarters of the experiment (all *p* > 0.189; see supplementary Section II for details, cf. Figure 8a). Most importantly, we compared proportions of orthogonal initial strokes of Experiment 2 and 3 (Fig. 8b) with two Bonferroni-corrected Welch-tests. Welch tests are an alternative to independent sample t-tests when sample sizes and -variances are unequal. For 0% quality trials, proportions did not differ between the two experiments, *t*(1,24.87) = 0.29, *p* = 1, *g* = 0.1. For 50% quality trials, proportions were significantly higher for Exp. 2 than for Exp. 3, *t*(1,19.81) = 2.6, *p* = 0.034, *g* = 0.85.

Finally, proportions of orthogonal middle and last strokes from Exp. 3 entered three-way mixed-design ANOVAs with the within-participants factors Visual Quality and Demand and the between-participants factor Order. In contrast to Exp. 2, results revealed no effect of Order, *F*(1,12) = 1.45, *p* = 0.25, η^2^_*p*_ = 0.10, but a main effect of the factor Demand, *F*(1,12) = 5.65, *p* = 0.035, η^2^_*p*_ = 0.32, suggesting higher adjustments when task demands are higher. There was again no main effect of Visual Quality, *F*(2,24) = 0.56, *p* = 0.595, η^2^_*p*_ = 0.01, and no interaction effect (all *p* > 0.07). For last strokes, no effect reached significance (all *p* > 0.12).

## Discussion

In the current study we aimed to clarify whether humans use visual priors for exploratory movement adjustments, enabling optimization of sensory information gathering. We also asked whether this behavior can be further moderated by the quality of the priors and the present task demands, and which mechanism is likely to enable the behavior. Experiment 1^13^ demonstrated that indeed, visual priors indicating a texture’s orientation produce orthogonal movement adjustments at initial contact similar to those that typically occur over the course of an exploration^12^. Additionally, it became evident that the higher the priors’ quality, the more pronounced the movement adjustments become. This is in line with a number of empirical findings and motor control models which suggest that sensory and prior information are weighted and used according to their reliability^28–30^. We conclude that this principle also applies to the usage of prior (visual) information for exploratory movement preparation. Our findings thus underline the dynamic and adaptive nature of active touch: When having access to prior visual information of sufficient quality, humans use it to employ more efficient exploratory strategies. Notably, only 2 out of 16 participants reported having intentionally used the visual priors, or at least having paid attention to them. This could seem surprising as the importance of active attention to the relevant signals in a task has been widely claimed in the field of perceptual learning^31,32^. However, several studies also provide evidence for the effectiveness of perceptual learning processes without the observer’s awareness and active attention^33,34^. Whether the process takes place despite the observers’ unawareness or whether their unawareness is required, remains an open question. This is particularly interesting as Zöller and colleagues observed force-tuning behavior in reaction to implicit prior information (from recurring presentation of stimuli) but found no clear effects for explicit semantic and visual information^7^. They argued that a reason for this could be the potential of explicit knowledge to interfere with automatic responses^35^. It might be worth investigating which behavioral consequences less abstract variants of visual priors such as highly naturalistic stimuli or even explicit instructions would provoke in our paradigm. Additionally, we observed an experience-dependent change of the amount of close-to-orthogonal movements in reaction to visual priors of high visual quality. During the first quarter of the experimental session, participants showed substantially less adjustment behavior as compared to the following quarter. This suggests that the underlying process is not fully based on pre-existing interpretations of the visual system but rather a learned behavior: (implicit) knowledge about the pairing of visual and haptic stimuli is gradually established, with the movement adjustments in reaction to the priors possibly being reinforced by a more convenient exploration experience.

In Experiment 2, we replicated the findings regarding the effect of the priors’ quality on their usage for exploratory movement adjustment. The focus however was on the potential effect of task demands: We hypothesized that higher task demands might be associated with a stronger usage of the priors for movement adjustments as a mean to compensate for impeded haptic discriminability^1,25^. When analyzing the difference between the low and high-demand trials from a within-participant perspective, we did not observe any substantial differences regarding the amount of orthogonal movements. Notably though, higher task demands were related to a higher number of total strokes, more changes between stimuli, and longer times spent on the stimuli; which most likely reflect compensatory mechanisms. Hence, the lack of effect is presumably not caused by an unsuccessful manipulation of the demands. Task demands thus seem to not have a rapidly modulating predictive effect on the use of the priors*.* Instead, we observed a trend suggesting that participants who were initially confronted with the more demanding block showed more pronounced adjustment behavior throughout the session. This would imply that tasks demands rather affect the learning process instead of the direct prior usage. The initial task demand was also related to an increased intake of sensory information, reflected by a higher proportion of orthogonal later strokes when participants began with the more demanding block. This has not been observed before and could underline the initial demand’s relevance for movement optimization during prolonged tasks; however it might be mainly caused here by the initial movement adjustments mentioned before. For an in-depth investigation of especially those initial movement adjustments, a between-participant design would be more feasible. Manipulating other task demands by e.g. restricting the exploration duration could also be of interest for this aim^36^. Surprisingly and in contrast to Experiment 1, the extent of movement adjustment did not substantially change over the course of the current experiment; the observed adaptation was mostly finalized within the first quarter of the session. Thus, the learning of adjustments seems to evolve rather quickly. The lack of time-dependency here could also be partly caused by the fact that half of the participants began the experiment with the more demanding block, in line with observed trend of increased adaptation behavior for this group. 

Interestingly, response accuracy remained constant across different conditions of visual prior quality in both the first and the second experiment. This might seem unexpected, as the prior usage should benefit perception in order to be deemed as behavior optimization. One reason for this lack of effect might be a ceiling effect, as response accuracy was relatively high overall and that we used a rather rough performance measure here only, and not a sensitive measurement of the just-noticeable-differences as in^12^. Another reason could be that the task involved a completely unrestricted haptic exploration, allowing participants to use other strategies to maximize performance. Still though, one might then suspect certain other parameters related to exploration efficiency to be affected by prior quality; such as the number of movements and changes between stimuli or the total time spent on the stimuli. Although this was also not reflected in the data, it is possible that exploration strategies differed in subtle and even individually different ways. Importantly, orthogonal movements have already been shown to improve perceptual precision^12^. Hence, the current study did not focus on explicitly assessing the merit of the prior usage in the context of task performance or exploration efficiency, but rather on investigating whether the presentation of visual priors elicit movement adjustments at all.

In Experiment 3, we investigated the role of the priors’ quality in the learning of adjustment behavior. For that, we presented lower- instead of the medium quality priors. As these lower-quality priors yielded the lowest informational value and have been shown produce the lowest adaptation behavior in Experiment 1, this manipulation can be construed as decreasing the contingency between visual priors and haptic information. The overall lower contingency was shown to deteriorate the establishment of movement adjustments: The results revealed no substantial movement adjustments as a reaction to the visual priors, reflecting an even stronger impairment of movement adaptation than previously expected. Direct comparisons showed that participants executed fewer close-to-orthogonal initial strokes during Exp. 3 than in Exp. 2. As the only methodological difference between the two experiments was the variation of quality conditions, we conclude that higher proportions of low-quality priors affect the learning process similar to how a lower contingency affects the learning of a pairing of two events^26^: a certain minimum contingency of priors and haptic stimuli seems crucial for the learning and thus for establishing adjustment behavior in the first place. This suggests that when a potential source of prior information does not yield sufficient informational value, this source is not used for the preparation of subsequent haptic exploration. This neglect possibly minimizes behavioral errors, as behavior is not adjusted according to e.g. ambiguous information. Especially because the visual priors in our paradigm are rather abstract and indirectly related to the upcoming stimulus as they yield information on its’ orientation but not the spatial frequency itself, the learning process might be sensitive to such changes in the overall contingency. Still, one could suspect a delayed, slower learning of the priors’ relevance in comparison to Experiment 1 and 2 and thus later onsets of adaptation behavior. However, the extent of adaptation behavior—or rather the lack thereof—did not change over the course of the session. This is in line with previous research: in^27^, the contingency learning effect disappeared when contingency was reduced to a certain threshold. The results on the one hand reemphasize that a learning process enables the adjustment behavior, as the usage of optimal priors should not be that strongly affected if mainly pre-existing interpretative processes would be the basis. For less abstract and more naturalistic stimuli, this might be different; plausibly, their presentation would provoke immediate adjustments of exploratory behavior. Mostly, the results underline the relevance of the priors’ quality: the disparity between priors of differing qualities regarding their value for our perceptual system and their assigned weight in the process of movement preparation became even more evident. One could have argued that when only considering the first experiment's results, low quality priors might actually evoke movement adjustments as well; but simply in a suboptimal way. This might yield the same effect on behavioral level (movement variability), but surely would have different implications. The last experiment demonstrates that at least before the adjustment behavior is established, priors differing in their quality seem to be in fact treated inherently different from each other. Whether this is still entirely true for the direct usage once the behavior is established, cannot be answered with utter certainty yet. It also remains open what the actual “shape” of movement adjustments looks like, i.e., whether the majority of movements tend to drift to the orthogonal axis or if some movements directly are adjusted onto the more or less orthogonal axis while some are not*.* Although we observed—unsurprisingly—no effect of task demands on the usage of priors, we found higher task demands to be associated with more pronounced adjustments for later movements in the last experiment*.* The observed immediate effects of task demand are in line with previous research showing that task demands evoke spontaneous exploratory movement optimizations^1^, directed at the intake of sensory information. This process thus seems to be more prone to be influenced by task demands than the more complex link between usage of priors which in turn improves the intake of sensory information.

## Conclusion

In the present study we demonstrated that humans autonomously learn to use rather abstract visual prior information for optimization of their haptic exploration process. It became evident that the learning process as well as the direct usage of the visual priors strongly depend on the priors’ quality. Specifically, the number of stroking movements orthogonal to a textures’ orientation was increased when visual priors indicated the orientation of the upcoming haptic stimulus; the more so the higher the priors’ quality. The effect of visual quality on the direct usage of the priors is in line with assumptions of motor control models and related empirical findings, as more reliable information is supposed to be given a higher sensory weight in the process of movement preparation and execution^20,21^. In order for the adjustment behavior to establish in the first place, a minimum contingency of high-quality visual priors and haptic stimuli appeared to be required. Unexpectedly, higher task demands did not increase the direct usage of the priors. Instead, the task demands that participants were initially confronted with seemed to possibly affect the establishment of adjustments as a whole. Due to the fast, but gradual establishment of prior usage and the fact that participants did not necessarily have to show subjective awareness^37^ about the priors’ purpose, the establishment of the prior usage appears to resemble a rather implicit learning process. Eventually, findings on how humans use prior information for exploratory movement control can be applied in the field of neurorobotics to improve object recognition or—manipulation of autonomous agents^38,39^. The optimization of exploration processes could improve information gathering, allowing for a more efficient and more accurate decision-making.

## Methods

### Experiment 1

#### Participants

Due to the very strong effect size reported for the movement adjustments over the course of an exploration of similar grating stimuli (d_z_ = 1.1)^12^, we expected a medium-to-large effect (f = 0.35) for the current experiment. Based on that, we conducted an a priori sample size calculation for a power of 80% and an alpha of 5%. The projected sample size was *N* = 14 for the within-participants factor of a repeated-measures ANOVA (G*Power,^40^). Hence, 16 right-handed students from Justus-Liebig University Giessen participated (10 female, mean age: 22.6 years, range: 18–27 years). Participants had normal or corrected-to-normal vision and reported no tenosynovitis in the past and no motor or cutaneous impairments. We confirmed that participants had no sensory deficits by conducting a two-point discrimination test; all had two-point discrimination thresholds lower than 3 mm on their index fingers^41^. They were naïve to the purpose of the experiment, provided written informed consent and received financial compensation (8€/hour). The experiment was approved by the local ethics committee Lokale Ethik-Kommission des Fachbereichs 06 (LEK-FB06) and conducted in accordance with 2013 Declaration of Helsinki, except for preregistration.

#### Setup and stimuli

Participants sat at a custom-made visuo-haptic workbench (Fig. 1a). It consisted of a PHANToM 1.5A haptic force feedback device (spatial resolution: 0.03 mm, temporal resolution: 1000 Hz; used only to track finger position), a force sensor to collect data of the normal force when the participant touches the stimulus (682 Hz, resolution: 0.05 N), and a 24" computer screen (120 Hz, 1600 × 900 pixel). Participants looked at the screen through a mirror while wearing stereo glasses (Nvidia 3D Vision 2, viewing distance 40 cm). The visual setup displayed 3D scenes aligned with the haptic workspace in front of the participant and prevented them from seeing their hand. Grating stimuli were displayed as light grey cylindrical discs on a dark green checkerboard. At the same positions two real stimuli were placed side-by-side with a force sensor underneath. A small sphere (diameter: 8 mm) represented the participants’ finger position in the scene. It disappeared while they touched the stimulus. A chinrest stabilized their head position. Via a spherical magnet fixed at the fingernail, the right index finger was connected to the PHANToM, allowing to move the finger in all axes with a maximum amount of freedom in a 38 × 27 × 20 cm^3^ workspace and leaving the fingertip free for bare-finger exploration. Devices were connected to a PC where C++—based custom software controlled the experiment and processed the data. Passive noise-cancelling headphones and white noise were used to mask any acoustic noises, The haptic stimuli consisted of four 3D-printed grating stimuli (Fig. 1b; printer: Stratasys Objet 30 Pro, resolution: 600 × 600 × 1600 dpi). We printed 4 mm high (z-axis) grating discs with a texture diameter of 90.7 mm (100.7 mm including the rim). A handle (10 × 5 mm) helped the experimenter arranging the stimuli before each trial. Each stimulus consisted of a groove pattern following a sine-wave function in height z (x):

$$z= \frac{1}{2}A\text{sin}\frac{2\pi x}{P}+\frac{1}{2}$$

Stimuli all had the same amplitude *A* of 0.3 mm and differed only in their period *P*. They had periods of 1.524 mm, 2.032 mm, 2.540 mm, and 3.048 mm. Stimuli with adjacent periods were compared, resulting in three pairs in total. Every pair was presented in 6 possible orientations relative to the observer (15°, 45°, 75°, 105°, 135° and 165°; for 0°, ridges would be parallel to the body, see Fig. 1b). As visual priors, visual representations of the grating stimuli were displayed on the screen with a texture made of 100 dark grey line segments (8 × 1 mm) on their top side (Fig. 1c). The textures indicated the orientation of the upcoming haptic gratings and were displayed before exploration. The quality of the priors was manipulated by varying the percentage of line segments following the same orientation as the gratings’ ridges while the remaining lines segment were randomly oriented. Pilot studies (N = 16 in total) had demonstrated that participants perceived the orientation of such stimuli excellently when 100% to 50% of the line segments were identically oriented. Variance in answers systematically increased only with lower percentages. Hence, we presented visual stimuli with percentages of 50% or lower.

#### Design and procedure

We varied the quality of prior visual information on stimulus orientation in five steps (50%, 35%, 25%, 15%, and 0% [= no information]). The haptic stimuli (and consequently the visual priors) were presented in 6 possible orientations (15°, 45°, 75°, 105°, 135° and 165°). Three different pairs of stimuli with adjacent periods were presented in both possible left–right assignments. Participants had to accomplish a two-alternative forced choice discrimination task: on each trial they explored two stimuli, starting equally often with the left and right stimuli, and had to decide which one had the higher spatial frequency. Overall, each participant conducted 360 trials in randomized order (5 qualities × 6 orientations × 3 pairs × 2 stimulus location × 2 start location). We implemented a break of 3 min every 60 trials. In total, the experiment took ca. 3.5 h. During each single trial, prior information was displayed for 2500 ms before participants were allowed to begin with the haptic exploration. The exploration was initiated by a beep sound. To indicate where participants should start, one of the two visual stimulus representation-discs was highlighted in yellow. They were instructed to use the typical movement scheme for the exploration, i.e. stroking over the surface^3^ and to switch between stimuli as often as desired. After the exploration they indicated which stimulus they had perceived to be of higher frequency by pressing a virtual button above it. White noise was presented through the headphones during the whole experiment to mask any exploration sounds. Before starting with the main experiment, participants performed eight test trials with 0% prior information textures and separate stimuli, which were not used in the main experiment, to familiarize themselves with the task. Participants filled out a questionnaire after the experiment which purpose was to check whether they had paid attention to the visual prior information and intentionally used it for movement adjustments. This contained three questions: (1) Did you actively pay attention to the visuals that were displayed at the beginning of each trial? (2) If yes, did you change your behavior in reaction to them and if so, how? (3) Did you feel discomfort at any point during the experiment? The last question served as a check-up for the feasibility of the design in general. Participants were not informed about the relationship between visual priors and the gratings’ orientation beforehand.

#### Data analysis

We analyzed movement directions of the initial, middle and last movement (“strokes”) in the exploration of each stimulus pair. A stroke was defined by a single unidirectional exploratory movement across the grating. In case of an even number of total strokes, the later of the two possible ones was defined as the middle stroke. A custom-written algorithm segregated single strokes from movement data when the participants’ finger was touching the stimulus area with at least 0.1N of force for > 200 ms. We detected strokes as continuous movements either from one texture border to another or between two movement turns, being extracted by zero crossings in the 1st order derivatives of the x- or z-position over time. Movement direction was determined as the orientation of the line connecting start and end point (x and z coordinates) of each stroke. Movement directions were processed as axial data and thus ranged only between 0°–180°. All stimulus orientations were aligned with 0° to collapse data over trials. We calculated circular histograms of initial movement directions (bin size: 10°) separately for each visual quality condition. Each histogram displays how many times participants moved in a specific direction. For statistical analyses, we focused on the initial strokes. Movement directions were analyzed using the MATLAB Circular Statistics Toolbox^42^: We used V-tests on the distributions of individual average movement directions per visual quality condition and separately for the initial, middle and last movements to test whether the distribution is not uniform (= all directions are equally likely) but rather has a specified mean direction of 90° (= optimal adaptation behavior). The middle and last strokes are analyzed mostly to allow for replication of previous findings^12^. Significant test statistics imply a deviation from uniformity in the suspected direction. Additionally, we compared proportions of close-to-orthogonal initial strokes (90° ± 15°) between quality conditions across participants using a repeated measures analysis of variance (ANOVA) with the within-participant factor Visual Quality. This range served as an indicator for a general drift of movement direction. The specific range has previously been used to detect movement changes over the course of an exploration^12^, and seemed feasible the current analysis as well, as we did not expect more distinct and precise movement adaptations in reaction to the priors than in reaction to gathered haptic sensory information and because we aimed for data that can be linked to those previous findings. A more conservative criterion (90° ± 10°) led to the same conclusions. Hence these results are not reported in the paper. To evaluate the establishment of movement adjustments over the course of the experiment, we focused on the percentage of close-to-orthogonal strokes when visual quality was very high (50%). We split data into the four chronological quarters of the experimental session (Trial 1–90, Trial 91–180, Trial 181–270, Trial 271–360) and analyzed it using a repeated measures ANOVA with the within-participant factor Quarter (Quarter 1–4). As we did not intent to conduct an in-depth analysis of the time course but were interested whether the adaptation behavior appears immediately or rather needs some time to get established, we decided to only analyze the condition that likely produced the most pronounced adaptation behavior, thus potentially revealing the strongest differences. Whenever the assumption of sphericity was violated, the *p*-values of the respective ANOVA were Greenhouse–Geisser adjusted^43^).

### Experiment 2

#### Participants

Following similar power considerations as for Experiment 1, 18 right-handed students from Justus-Liebig University Giessen participated (14 female, mean age: 23.05 years, range: 19–28 years.). They were naïve to the purpose of the experiment and had not participated in the previous experiment.

#### Setup and stimuli

The setup was identical to that of Experiment 1. Here we used 8 haptic stimuli in total, resulting in 4 pairs (two for each demand condition). The two “low demand” pairs were defined by a difference of ~ 0.5 mm between their periods (ca. 2 Weber fractions^44^): Their stimuli had periods of 1.524 mm versus 2.032 mm and 2.540 mm versus 3.048 mm. “High demand” pairs had a difference of ~ 0.25 mm between their periods (ca. 1 Weber fraction): 1.92 mm versus 2.16 mm and 2.40 mm versus 2.64 mm. The mean of all periods of the “high demand” stimulus set was the same as the one of the “low demand” stimulus set and all periods of one set were equidistant to each other.

#### Design and procedure

For economic reasons, as visual quality was not the main focus anymore, the experiment included only the three quality conditions 0%, 25% and 50%. We chose these levels as they were equidistant to each other with regards to the percentage of line segments following the same direction as the upcoming grating. Additionally, the design included two levels of task demand. The level of task demand was established implicitly by presenting trials of the same condition in one block, with 4 alternating blocks in total (ABAB/BABA design). The number of sessions starting with the “low demand” and the “high demand” condition was counterbalanced and randomized across participants. The instructions, task and procedure during the trials were the same as in Experiment 1. Each of the two demand conditions consisted of 144 trials in random order (3 qualities × 6 orientations × 2 pairs × 2 start position × 2 stimulus position), so that each participant conducted 288 trials in total. The experiment took on average 2.5 h and breaks were implemented after every block (72 trials).

#### Data analysis

Movement directions were extracted in the same way as described for Experiment 1. We also did the same analyses on these data as in Experiment I, except for that the overall ANOVAs on close-to-orthogonal strokes included three factors: the within-participant factors Visual Quality (0%, 25%, 50%) and Task Demand (low/high) and the between- participant factor Order (low first/hard first), to explore whether there is a difference between participants that started the experiment with the low versus the high demand block. Although the trial number per quarter was not the same as in Exp. 1, we again split data into the four chronological quarters of the experimental session and conducted the same ANOVA, as we aimed to check for a general trend rather than to compare exact time courses between experiments. If anything, we would expect higher differences especially between the first and last quarter due to the smaller number of trials in this experiment and consequently its’ first quarter.

### Experiment 3

#### Participants

Exp. 3 focused on studying prior learning in comparison to Exp. 2. Starting from the strong effect of the priors’ quality on their direct usage found in Experiment 2, we expected at least medium-sized effect of the priors’ quality on the prior learning process (f = 0.25). Based on that, we conducted an a priori sample size calculation for a power of 80% and an alpha of 5%. The projected sample size was 28 for the within-between interaction of a repeated-measures ANOVA^40^. We decided for a sample size of *N* = 14 in addition to the *N* = 18 of Experiment 2. Accordingly, 14 right-handed students from Justus-Liebig University Giessen participated (9 female, mean age: 25.29 years, range: 19–29 years). They were naïve to the experiment’s purpose and had not participated in the previous experiments.

#### Design and procedure

The setup, stimuli and procedures were similar to those of Exp. 2 except that we used the visual quality conditions 0%, 15%, and 50% (instead of 0%, 25%, and 50% as in Exp. 2). We chose the 15% condition here, as it was the lowest of the previously implemented quality conditions and in line with that, produced the lowest adaptation behavior in Experiment 1.

#### Data analysis

We applied the same data analyses as for Exp. 2. In addition, we compared proportions of orthogonal initial strokes of Experiment 2 (high contingency) and 3 (low contingency) separately for 0% and 50% quality-trials with two Bonferroni-corrected Welch-tests. We used Welch-tests instead of checking for an interaction effect with an ANOVA here in order to account for unequal sample sizes and -variances.

### Supplementary Information

Supplementary Information.

## Data Availability

The datasets generated and analyzed during the current study are available at 10.5281/zenodo.7639118.
