# Supplementary material for: Humans flexibly use visual priors to optimize their haptic exploratory behavior
Source: Sci Rep. 2024 Jun 28;14:14906. doi: 10.1038/s41598-024-65958-6 (PMC11213930; doi:10.1038/s41598-024-65958-6)
Supplement: Supplementary file 1 — Supplementary Information. [file 41598_2024_65958_MOESM1_ESM.pdf]

Supplementary Information

Humans flexibly use visual priors to optimize their haptic exploratory behavior

Michaela Jeschke, Aaron C. Zoeller, Knut Drewing

Section I

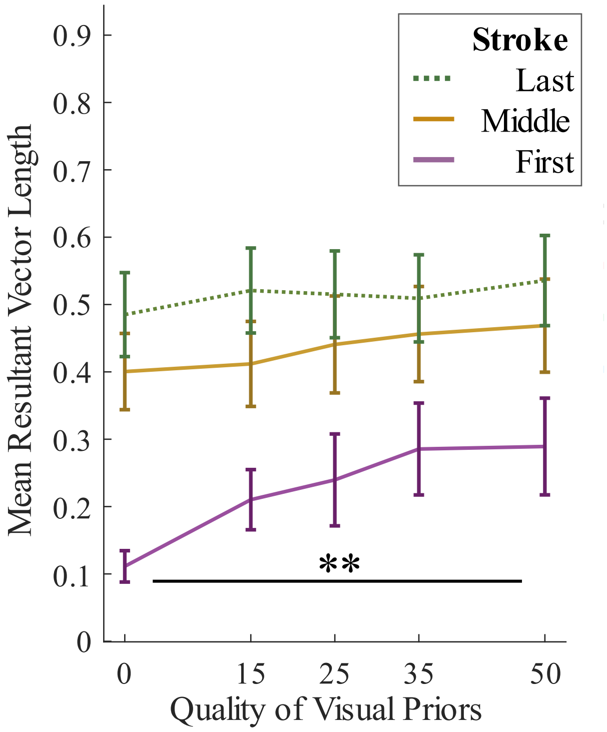

Supplementary Figure S1. Average mean resultant vector lengths and standard errors for each quality condition and movement type.

In Experiment 1, the mean resultant vector length $\bar{R}$ of each participants' initial movement directions for each visual quality condition entered a repeated measures ANOVA to compare the variance of movement directions between quality conditions. $\bar{R}$ is the vector length of the mean direction $\bar{\theta}$ of a circular distribution. It varies between zero and one; an $\bar{R}$ near one implies that there is little variation in movement directions and the data is concentrated around the mean direction $\bar{\theta}$. Vectors were longer with higher visual qualities, i.e. variability of the initial movements' directions decreased with higher visual quality, $F(4, 60) = 4.81$, $p = .03$, $\eta^2 = 0.24$ (Greenhouse-Geisser adjusted), confirmed by a linear trend, $F(1,15) = 6.16$, $p = .025$, η^2_p =

0.30. For middle and last strokes, there was no effect of visual quality, $F(4, 60) = 2.22$, $p = .08$, $\eta^2 = 0.13$, and $F(4, 60) = 1.74$, $p = .15$, $\eta^2 = 0.10$, respectively.

Section II

In Exp. 3, proportions of orthogonal initial strokes did not differ between visual qualities, $F(2, 24) = 0.58$, $p = .568$, $\eta^2_p = 0.04$, nor did the results reveal a main effect of Task Demand, $F(1, 12) = 0.05$, $p = .833$, $\eta^2_p = 0.01$, or a main effect of Order, $F(1,12) = 1.05$, $p = .325$, $\eta^2_p = 0.02$. Similarly, no interaction effect reached significance (all $p > .189$). Proportions of orthogonal initial strokes of trials with high visual quality entered a one-way repeated measures ANOVA with the within-participants factor Quarter. No main effect of Quarter was observable, $F(3, 39) = 0.21$, $p = .891$, $\eta^2_p = 0.02$, (Quarter 1: $M = 21.12$, $SD = 7.20$, 2: $M = 21.41$, $SD = 9.48$, 3: $M = 22.90$, $SD = 10.19$, 4: $M = 23.29$, $SD = 10.71$).
